# Optimizing fluidity and tensile strength of magnetically driven epoxy-cement repair materials based on response surface

**DOI:** 10.1038/s41598-023-36233-x

**Published:** 2023-06-14

**Authors:** Shifu Qin, Jie Liu, Xiaoping Wang, Fan Yu, Zheng Li, Delin Tan, Qiao Yan, Lehua Wang

**Affiliations:** 1 College of Civil Engineering and Architecture, China Three Gorges University, Yichang, 443002 China; 2 Key Laboratory of Geological Hazards On Three Gorges Reservoir Area (China Three Gorges University), Ministry of Education, Yichang, 443002 China; 3 College of Hydraulic and Environment, China Three Gorges University, Yichang, 443002 China

**Keywords:** Civil engineering, Structural materials

## Abstract

Underwater crack repair is challenging due to drainage and exhaust, slurry retention at fixed points, and other issues. Magnetically driven epoxy resin cement slurry was developed, which can perform directional movement and fixed-point retention of slurry under the effect of an applied magnetic field. This paper focuses on slurry fluidity and tensile properties. Firstly, in the preliminary pre-study, the main influencing factors of the ratios were determined. Then, the optimum range of each factor is determined by a single-factor experiment. Furthermore, the response surface method (RSM) is applied to obtain an optimal ratio. Finally, the slurry is characterized by micro. Results showed that the evaluation index F proposed in this paper can well evaluate the interaction between fluidity (*X*) and tensile strength (*Y*). The 2FI regression model and the quadratic regression model are developed with fluidity and tensile strength as the response values and Epoxy Resin (ER) content, water-cement ratio, Fe_3_O_4_ content and sulphoaluminate cement (SAC) content as the influencing factors, and have reasonable fit and reliability. The relationship between the degree of influence of the influencing factors on the response value *X* and the response value *Y* in ascending order was: ER content > water-cement ratio > SAC content > Fe_3_O_4_ content. The magnetically driven slurry made by the optimal ratio can reach a fluidity rate of 223.31 mm and a tensile strength of 2.47 MPa. This is with relative errors of 0.36% and 1.65% from the model predicted values. Microscopic analysis showed that the magnetically driven epoxy resin cement slurry had a favorable crystalline phase, surface morphology, and structural composition.

## Introduction

With the rapid growth of the global construction industry, a large number of infrastructures are being planned and being constructed. Many underwater concrete structures in service are susceptible to cracks and holes due to freeze–thaw cycles^1–3^, dry–wet cycles^4,5^, sulfate and chloride erosion^4,6,7^, resulting in significant deterioration of their performance^8^. Although, repair materials in the building field, polymer-modified cementitious materials have been widely applied^9,10^. However, the repair and reinforcement of underwater concrete structures must face the problems of construction drainage and exhaust, upward sloping cracks and defects, low filling rate of tiny fissures, and difficult slurry retention under moving water conditions, which makes this repair work still challenging^11^.

**Figure 1:**
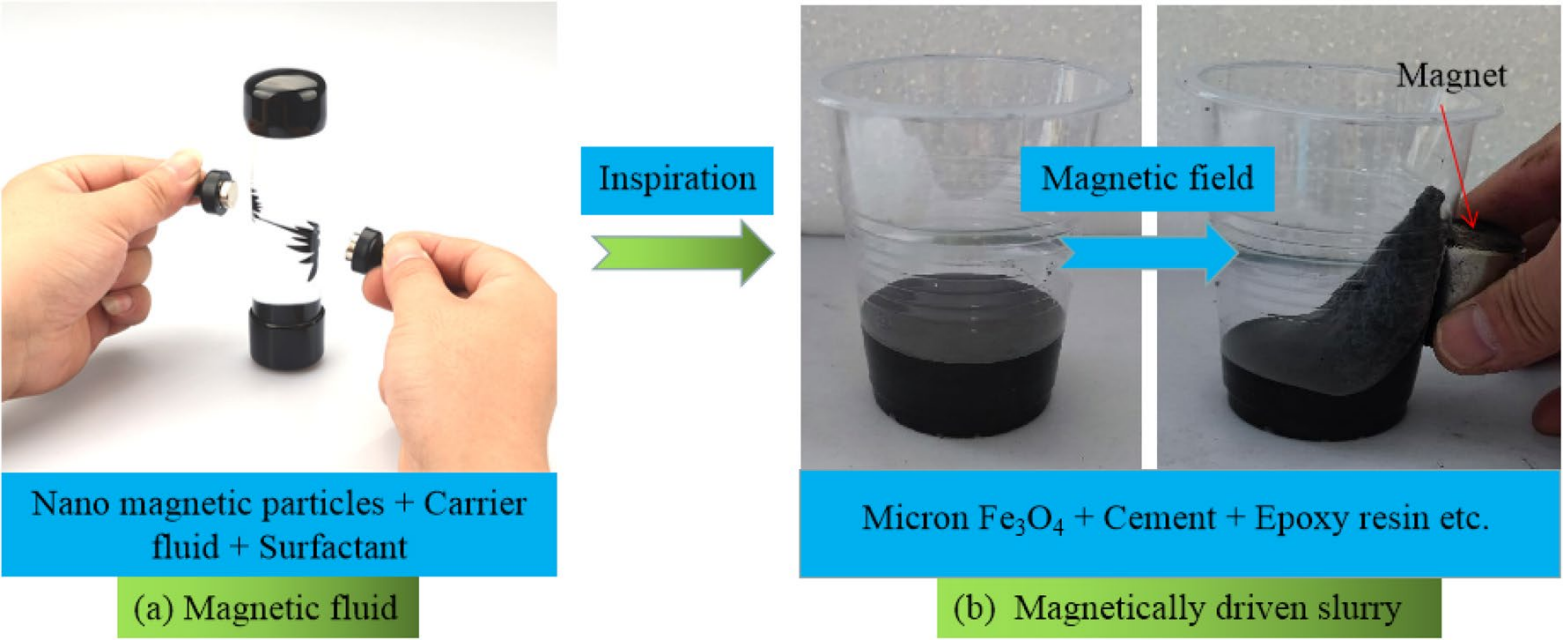
Schematic diagram of magnetically driven epoxy-cement slurry.

At present, conventional pressure grouting methods cannot solve the problems of ventilation, drainage, and slurry retention at fixed points. Inspired by magnetic fluids, we are developing a magnetically driven epoxy resin cement slurry. This will achieve directional movement and fixed-point retention under an applied magnetic field, as shown in Fig. 1. This work is based on the principle that Fe_3_O_4_ can be "target-driven" under a magnetic field^12^. The magnetically driven slurry with fresh properties of the slurry possessed the capacity to fill, move and resist segregation^13–15^, which can overcome gravity to repair upward sloping cracks and defects^16^. Liu et al.^16^ developed a magnetic epoxy resin cement grouting anchor material with anti-gravity self convergence, guided flow, and real-time controllable slurry viscosity under the action of magnetic field, and explored the mechanism of slurry hardening and microscopic pore change law under the action of magnetic field, without involving the study of fluidity and tensile strength properties of the grouting material. Fluidity determines slurry's diffusion ability and pumpability, which are key indicators of grouting construction performance^17^^.^ The tensile strength of the slurry curing material is intended to support the strength of grout repair plus solids^18^. However, as tensile strength increases, slurry fluidity often decreases^19^. The preliminary pre-study found that with the changes in ER content, water-cement ratio and Fe_3_O_4_ content, the changes in slurry fluidity and tensile strength behaved opposite. Consequently, an optimal design of the ratio of repair materials using relevant experimental design methods is required to obtain an optimized ratio. This is to well balance the fluidity and tensile strength of the magnetically driven epoxy resin cement slurry.

RSM is a reliable design method for experimental optimization, which integrates experimental design and mathematical modeling^20^. It can fit a regression model between each influencing factor and the response value. This will enable it to find the optimal combination of each factor and the optimal value of the response target^21^. There are fewer experiments, a shorter timeframe, high precision, reliable prediction results, and the interaction between factors can be studied with it^22^.

Therefore, the fluidity and tensile strength of the slurry are studied in this paper. The regression models are established by the RSM to obtain the optimal mix proportions and the corresponding response values. Firstly, the combined effect evaluation index *F* of fluidity and tensile strength is introduced. The influence of ER content, water-cement ratio, Fe_3_O_4_ content and SAC content on *F* is investigated by single-factor experiments, and the level range of each factor is measured. Then, the 2FI regression model and quadratic regression model are established by central-composite design(CCD) RSM, with fluidity and tensile strength as response values, and ER content, water-cement ratio, Fe_3_O_4_ content and SAC content as influencing factors. In this step, model variance and response surface analysis are used to determine the model fitting effect and the influence of each factor interaction on the response value. Furthermore, optimization analysis is performed using design experts to obtain the optimal mix proportions and the predicted value of the response value. The cement slurry with these mix proportions is formulated, and the measured value of the response value was obtained by tensile and fluidity experiments to obtain the relative error. Finally, the solidification of the magnetically driven epoxy resin cement slurry with optimal mix proportions is characterized by X-ray diffraction (XRD), scanning electron microscope (SEM), and Fourier transform infra-red (FTIR).

## Experimental

### Raw materials

Commercial waterborne epoxy resin was produced from Shenyang Dongyan Tuyan Decoration Co., Ltd. (Shenyang, China), the relevant indicators are shown in Table 1. Fe_3_O_4_ with a density of 5.17 g/cm^3^, a specific surface area of 50 m^2^/g, and a purity of 99.9% with a powder diameter of 45 μm was provided by Hebei Casting & Research Alloy Materials Co., Ltd (Shijiazhuang, China) to improve the magnetic attraction of aqueous magnetically driven epoxy resin cement slurry. Hydroxyethyl methyl cellulose (also known as flocculant) was produced by Zhengzhou An Anankang Food Chemical Co., Ltd. (Zhengzhou, China) to enhance the dispersion resistance of the slurry. SiKa ViscoCrete-540P was used as a magnetically driven epoxy resin cement slurry water reducer, and the silicone antifoaming agent (also known as defoamer) was both used in this study.

**Table 1:** Component performance index in waterborne epoxy resin.

Composition	Appearance	Solid content /%	Density/(g/cm^3^)	Epoxy value (mol/g)	Activated hydrogen equivalent/(mol/g)	Viscosity (25 °C)/(mPa s)	PH
Epoxy resin	Creamy white	50 ± 3	1.05	0.52	–	11,190	6–8
Hardener	Pale yellow	49	1.05–1.08	–	330	6500–9000	9.5–10.5

Type P·O 52.5 ordinary Portland cement (OPC), SAC and silica fume (SF) were used as binders. The partial replacement of OPC with SAC is to shorten the coagulation time of the slurry forming the stone body. In addition, the partial replacement of OPC with SF is to improve the impermeability of the stone body^23^. The specific surface area of OPC, SAC and SF was 0.382 m^2^/g, 0.402 m^2^/g and 19.8 m^2^/g respectively, and their corresponding chemical compositions are shown in Table 2.

**Table 2:** Chemical composition of cement and silica fume used in this study (% by mass).

Material	SiO_2_	CaO	Al_2_O_3_	Fe_2_O_3_	MgO	SO_3_	Loss on ignition
OPC	20.86	56.77	5.9	3.61	3.5	2.43	1.16
SAC	9.96	41.89	28.91	3.18	1.7	8.97	0.49
SF	94.8	1.86	0.81	0.08	0.65	–	4.65

### Preparation of magnetically driven epoxy resin cement slurry

Emulsion type epoxy resin was obtained by mixing the epoxy resin composite with the waterborne hardener (hardener: pure epoxy resin at 0.85:1). magnetically driven epoxy resin cement slurrys were then prepared with a constant epoxy resin content (5% by mass of the total cement). The SAC:OPC ratio by mass was 10%:90%, and SF-to-cement was 0.05. For basic proportions, a fixed Fe_3_O_4_-to-cement ratio, water-to-cement and flocculant-to-cement ratios were applied, which were 1:5, 0.5 and 0.01 respectively. The defoamer (1 wt% of the epoxy resin aqueous solution) and the superplasticizer (denoted as SP with a fixed ratio of 1 wt% of the total cement) were also used for minimising bubbles introduced by the addition of epoxy resin and for improving the workability of the slurry, respectively. In order to show the relative contents of different components clearly, basic proportions are shown in Table 3.

**Table 3:** The basic proportions of magnetically driven epoxy resin cement slurry.

Cement		Water	Waterborne epoxy resin		Fe_3_O_4_	SF	Defoamer	Flocculant	SP
OPC	SAC		ER	Hardener					
90	10	50	5	4.25	20	5	0.0925	1	1

The total cement content is fixed as the value of 100 for the convenience of reading.

For the specimens, SF, SAC, and OPC were mixed for 30 s at low speed. Then Fe_3_O_4_ and flocculant were added into the mixing bowl. This dry mixture was mixed for 60 s. The defoamer, superplasticizer, water, epoxy resin and hardener were fully mixed for three minutes at the same speed before casting. The above aqueous solution must be prepared within 20 min prior to its addition into the dry powder system. This is in order to prevent its early hardening after the hardener has been was cast into dumbbell moulds. After 24 h, specimens were demoulded and cured in standard curing conditions (temperature = 20 ± 1 °C, relative humidity ≥ 95%) for six days. Forming and curing regimes were based on Chinese standards SL/T 807-2021 and DL/T 5126-2001, used for testing epoxy resin grout for hydraulic structures and polymer-modified cement mortar, respectively.

In order to study the effect of OPC replacement with SF, the fluidity and tensile tests of the reference group (0 of SF content) of "the basic proportions of magnetically driven epoxy resin cement slurry" were conducted. The fluidity of the basic proportions and reference groups was 237 mm and 234 mm, respectively. The tensile strengths were 1.36 MPa and 1.38 MPa, respectively. The results showed that replacement of OPC with 5% SF had no effect on the fluidity and tensile strength of the magnetically driven epoxy-resin cement slurry.

### Experimental design

#### Single factor experimental design

According to the previous experiments, ER content, water-cement ratio, Fe_3_O_4_ content and SAC content were determined as the main experimental influencing factors. On the premise of other conditions remaining unchanged, the ER content, water-cement ratio, Fe_3_O_4_ content and SAC content were changed in turn, and their effects on fluidity and tensile strength were studied to determine the optimal range of each factor.

#### Response surface optimization experimental design

The three-level, four-factorial Central-Composite experimental design with a categoric factor of 0 was employed to optimize the mix proportion based on the response values *X* and *Y*. The design was composed of three levels (high, medium and low, being coded as + 1, 0 and − 1) and a total of 30 runs were carried out in duplicate to optimize the level of chosen variables, such as ER content, water-cement ratio, Fe_3_O_4_ content and SAC content. For the purpose of statistical computations, the four independent variables were denoted as *A*_1_, *A*_2_, *A*_3_ and *A*_4_, respectively. According to the preliminary experiments, the range and levels used in the experiments are selected and listed in Table 4.

**Table 4:** Factors and levels of central-composite design (CCD).

Variables	Real values of coded levels		
	1	0	− 1
ER content, *A*_1_ (%)	9	7	5
Water-cement ratio, *A*_2_	0.55	0.5	0.45
Fe_3_O_4_ content, *A*_3_ (%)	20	15	10
SAC content, *A*_4_ (%)	7.5	5	2.5

The experimental design matrix of the Central-Composite Design is tabulated in Table 5, and corresponding experiments were performed. The results were analyzed by applying the coefficient of determination (R^2^), analysis of variance (ANOVA), contour lines, and response surface.

**Table 5:** Response surface experimental design and results.

Standard order	Factor				Tensile strength (MPa)	Fluidity (mm)
	ER content (%)	Water-cement ratio	Fe_3_O_4_ content (%)	SAC content (%)		
1	5	0.45	10	2.5	1.87	245.00
2	9	0.45	10	2.5	2.57	226.00
3	5	0.55	10	2.5	0.82	266.00
4	9	0.55	10	2.5	1.43	255.00
5	5	0.45	20	2.5	1.85	238.00
6	9	0.45	20	2.5	1.65	216.00
7	5	0.55	20	2.5	1.33	264.00
8	9	0.55	20	2.5	1.26	244.00
9	5	0.45	10	7.5	1.21	233.00
10	9	0.45	10	7.5	1.78	221.00
11	5	0.55	10	7.5	0.98	243.00
12	9	0.55	10	7.5	1.63	248.00
13	5	0.45	20	7.5	1.12	233.00
14	9	0.45	20	7.5	1.31	217.00
15	5	0.55	20	7.5	1.57	255.00
16	9	0.55	20	7.5	2.03	242.00
17	3	0.5	15	5	1.01	255.00
18	11	0.5	15	5	1.98	209.00
19	7	0.4	15	5	1.86	204.00
20	7	0.6	15	5	1.14	259.00
21	7	0.5	5	5	1.66	247.00
22	7	0.5	25	5	1.93	229.00
23	7	0.5	15	0	1.34	243.00
24	7	0.5	15	10	1.37	228.00
25	7	0.5	15	5	2.05	235.00
26	7	0.5	15	5	1.98	235.00
27	7	0.5	15	5	2.18	235.00
28	7	0.5	15	5	2.12	235.00
29	7	0.5	15	5	2.09	235.00
30	7	0.5	15	5	2.18	235.00

### Characterisation of magnetically driven epoxy resin cement slurry

#### Tensile strength test

**Figure 2:**
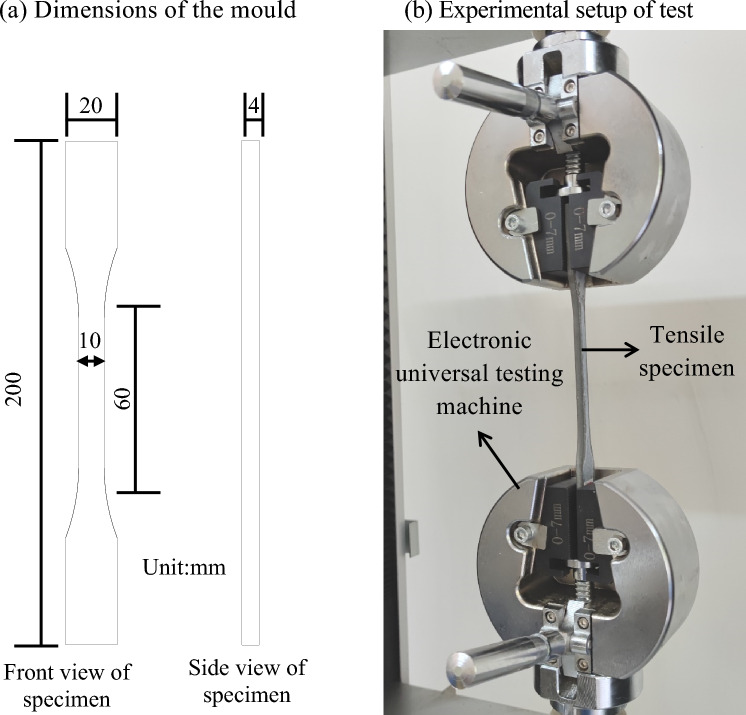
(**a**) The dimensions of the mould used and (**b**) the experimental setup for the tensile strength test of magnetically driven epoxy resin cement slurry.

Tensile strength test, in accordance with the Chinese standard (SL/T 807-2021) was carried out on dumbbell-shaped samples with the moulds specifically designed to fabricate the samples required for testing using an electronic universal testing machine, as shown in Fig. 2. The moulds were internally coated with some debonding agent in order to avoid the adhesion of the slurry to the mould during the curing process. For the tensile strength test, three samples were used and the average value was considered as the final result, and the measurements were performed at a speed rate of 0.5 mm/min.

#### Fluidity

The fluidity of magnetically driven epoxy resin cement slurry was assessed using a truncated cone fluidity test. The fluidity was obtained by averaging the diameters (mm) measured in three directions in accordance with the Chinese standard GB/T 50448-2015.

#### Microscopic analysis

The prepared specimens of optimal mix proportions were cured under standard curing conditions for 28 days. The crystal phase of solidification was determined by a Japan SmartLab diffractometer. A scanning electron microscope JSM-7500 F was used to measure the surface morphology of solidification. The structure composition of the solidified slurry was determined by a Fourier Transform infrared spectrometer.

## Single factor experimental results

### Effect of ER content

**Figure 3:**
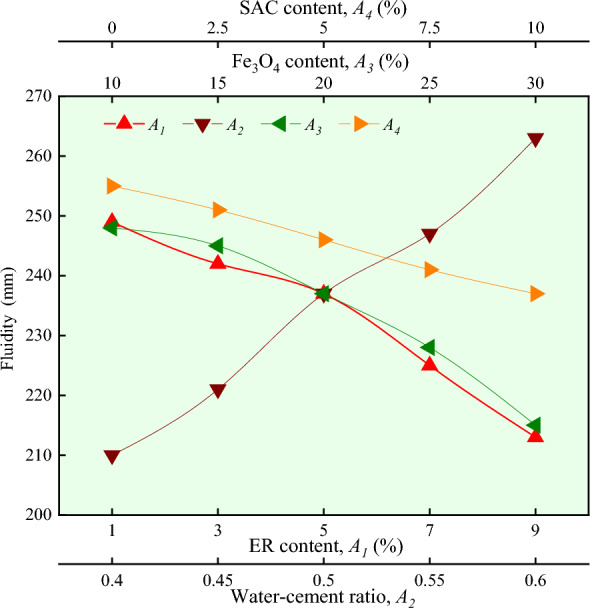
Effect of different factors on fluidity.

**Figure 4:**
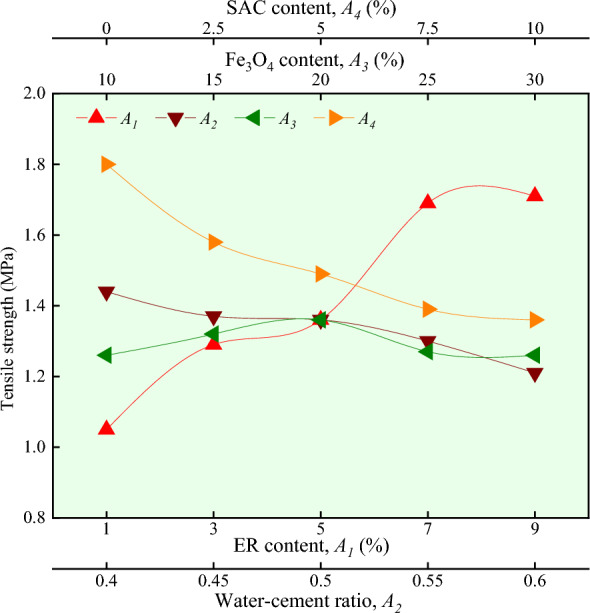
Effect of different factors on tensile strength.

It can be seen in Figs. 3 and 4 that with the increase of ER content, the value of degree of fluidity gradually decreases, while the value of tensile strength gradually increases. The greater the degree of ER content, the higher the viscosity, and the higher the viscosity, the smaller the fluidity^24^. This indirectly explains the uniform dispersion of ER in the magnetically driven epoxy resin cement slurry. As long as ER is cured, it can be used as a binder to bond the hydrated inorganic crystalline products to the cement paste. It can be seen from the SEM that the complexes are covered on the surface of the hydrated products or adsorbed on the inorganic surface. In some cases, it enhances the bonding ability of cement slurry solidification, thereby improving its tensile strength^19^.

A common evaluation index *F* such as Eq. (1) was introduced to evaluate the interaction effect of ER content on the value of degree of fluidity and the value of tensile strength of magnetically driven epoxy resin cement slurry.

1 $$ F = X \times Y $$

**Figure 5:**
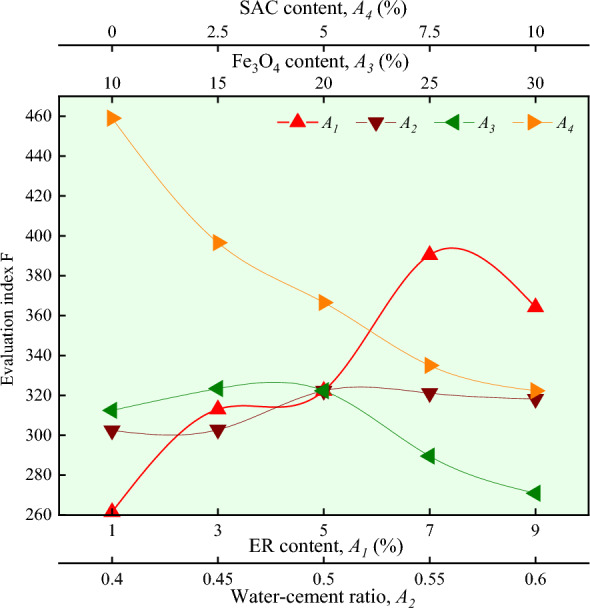
Effect of different factors on evaluation index *F.*

The evaluation index *F* under different ER content is shown in Fig. 5. As shown in Fig. 5, the evaluation index *F* increases and then decreases as the ER content increases, and the evaluation index *F* is maximum when the ER content is 7%.

### Effect of water-cement ratio

It can be seen in Figs. 3 and 4 that with the increase of water-cement ratio, the value of degree of fluidity gradually increases, while the value of tensile strength gradually decreases. At higher water-cement ratio, slurry has less consistency, poor cohesion and water retention, and in the same ER content, fluidity increases with the increase in water-cement ratio. In the same ER content, with the increase of water-cement ratio, resulting in more water, the pore space of the slurry increases, the structure is more loose, and the tensile strength is reduced.

Equation (1) is applied to calculate the evaluation index *F* under different water-cement ratios, and the results are shown in Fig. 5. It can be seen from Fig. 5 that as the water-cement ratio increases, the evaluation index F first decreases slightly, then increases and then decreases slowly, and the evaluation index *F* is maximum when the water-cement ratio is 0.5.

### Effect of Fe_3_O_4_ content

It can be seen in Figs. 3 and 4 that with the increase of Fe_3_O_4_ content, the value of degree of fluidity gradually decreases. In contrast, the value of tensile strength increases slightly and then decreases and then remains unchanged. Under the same conditions, due to the high density of Fe_3_O_4_, with the increase in Fe_3_O_4_ content, the degree of consistency of magnetically driven epoxy resin cement slurry increases continuously^17^. This results in the decrease of fluidity. Micro Fe_3_O_4_ can fill the pores of cement slurry. With an increase in Fe_3_O_4_ content, the structure is closer and the tensile strength is also improved. Tensile strength is highest when the content reaches 20%, according to some research^25^.

Equation (1) is applied to calculate the evaluation index *F* under different Fe_3_O_4_ content, and the results are shown in Fig. 5. It can be seen from Fig. 5 that with the increase in Fe_3_O_4_ content, the evaluation index *F* increases slightly and then decreases. The evaluation index *F* is maximum when the Fe_3_O_4_ content is 15%.

### Effect of SAC content

It can be seen in Figs. 3 and 4 that as the SAC content increases, the value of degree of fluidity gradually decreases. In contrast, the value of tensile strength decreases rapidly and then slowly. Under the same conditions of water-cement ratio, the flow of slurry is mainly related to the fineness of cement particles. The finer the particle size and the larger the surface area, the more water is required and the smaller the fluidity^26^. The particle fineness of SAC is larger than that of OPC, and as the SAC content increases, the fluidity decreases. Tensile strength is related to the setting time of magnetically driven epoxy resin cement slurry. As the SAC content increases, the CA_3_S content increases. The magnetically driven epoxy resin cement slurry hydrates faster and has a shorter setting time. Since the main hydration products are coarse crystals and cannot be evenly distributed over time, internal micro-cracks result in weak points, resulting in reduced tensile strength^27^.

Equation (1) is applied to calculate the evaluation index *F* under different SAC content, and the results are shown in Fig. 5. It can be seen from Fig. 5 that with the increase of SAC content, the evaluation index *F* gradually decreases. The SAC content of 5% is selected as the optimum considering the contribution to the shortening of the setting time.

## Response surface optimization analysis

For RSM, the most commonly used second-order polynomial equation and 2FI equation developed for regression fitting experimental data and determining the relevant model terms can be written respectively as:

2 $$ X = \alpha_{0} + \sum {\alpha_{i} } A_{i} + \sum {\alpha_{ij} } A_{ij} + \varepsilon_{X} $$

3 $$ Y = \beta_{0} + \sum {\beta_{i} } A_{i} + \sum {\beta_{ij} } A_{ij} + \sum {\beta_{ii} } A_{i}^{2} + \varepsilon_{Y} $$

where *X* and *Y* represent the predicted response, i.e. the fluidity (mm) and tensile strength (MPa) by the mix proportion optimization, α_*0*_ and β_*0*_ the constant coefficients, α_*i*_ and β_*i*_, the *i*th linear coefficient of the input factor A_*i*_, α_*ii*_ and β_*ii*_, the *i*th quadratic coefficient of input factor A_*i*_, α_*ij*_ and β_*ij*_, the different interaction coefficients between input factors A_*i*_ and A_*j*_ (*i* = 1–4, *j* = 1–4 and *i* ≠ *j*), ε_*X*_ and ε_*Y*_, the error of the model.

The equation expresses the relationship between the predicted response and independent variables in coded values according to Tables 4 and 5.

### Model equation

The fluidity and tensile strength experiments are conducted according to the design matrix and the corresponding results are listed in Table 5. The second-order polynomial equation and 2FI equation for predicting the optimum point were obtained according to the Central-Composite design and input variables^28^, and then the empirical relationship between the response and the independent variables in the coded units was presented on the basis of the experimental results as follows:

4 $$ X = 238.67 - 7.83A_{1} + 12.42A_{2} - 2.58A_{3} - 3.92A_{4} + 2.13A_{1} A_{2} - 2.25A_{1} A_{3} + 3.13A_{1} A_{4} + 2.00A_{2} A_{3} - 1.62A_{2} A_{4} + 1.50A_{3} A_{4} $$

5 $$ \begin{gathered} Y = 2.10 + 0.20A_{1} - 0.16A_{2} + 0.02A_{3} - 0.05A_{4} + 0.02A_{1} A_{2} - 0.13A_{1} A_{3} + 0.05A_{1} A_{4} + \\ 0.18A_{2} A_{3} + 0.24A_{2} A_{4} + 0.06A_{3} A_{4} - 0.15A_{1}^{2} - 0.15A_{2}^{2} - 0.08A_{3}^{2} - 0.19A_{4}^{2} \\ \end{gathered} $$

### Model variance analysis

#### Model variance analysis of the response value *X*

Regression analysis is performed on the response values *X* from Table 5, and the model ANOVA results for the response values *X* are obtained as shown in Table 6.

**Table 6:** Variance analysis results of the response value *X.*

Source	S_*S*_	D_*F*_	M_*S*_	F	P_r_ > F	Note
Model	7400.88	10	740.09	44.49	< 0.0001	**
*A*_1_	1998.38	1	1998.38	120.12	< 0.0001	**
*A*_2_	4187.04	1	4187.04	251.68	< 0.0001	**
*A*_3_	287.04	1	287.04	17.25	0.0005	**
*A*_4_	330.04	1	330.04	19.84	0.0003	**
*A*_1_*A*_2_	150.06	1	150.06	9.02	0.0073	**
*A*_1_*A*_3_	175.56	1	175.56	10.55	0.0042	**
*A*_1_*A*_4_	95.06	1	95.06	5.71	0.0273	*
*A*_2_*A*_3_	68.06	1	68.06	4.09	0.0574	
*A*_2_*A*_4_	33.06	1	33.06	1.99	0.1748	
*A*_3_*A*_4_	76.56	1	76.56	4.60	0.0451	*
Residual	316.09	19	16.64			
Lack of fit	316.09	14	22.58		0.0691	
Pure error	0.00	5				
Total	7716.97	29				

*S*_S_ is sum of squares, *D*_F_ is degrees of freedom, *M*_S_ is mean square, *P*r > F is probability without significant, *P*r > *F* ≤ 0.01 is extremely significant(**), *P*r > *F* ≤ 0.05 is significant(*).

As can be seen from Table 6, P_r_ > F < 0.000 1 is very significant, indicating that the model can be well optimized mix proportion. The lack of fit (P_r_ > F = 0.0691 > 0.05) is not significant, indicating that the model is significantly reliable. The F-test shows that the magnitude of the influence factor on the response value *X* is *A*_2_ > *A*_1_ > *A*_4_ > *A*_3_*. A*_1_*A*_2_ and *A*_1_*A*_3_ (P_r_ > F < 0.01) had a highly significant effect, *A*_1_*A*_4_ and *A*_3_*A*_4_ (P_r_ > F < 0.05) had a significant effect, while *A*_2_*A*_3_ and *A*_2_*A*_4_ (P_r_ > F > 0.05) had a non-significant effect.

**Figure 6:**
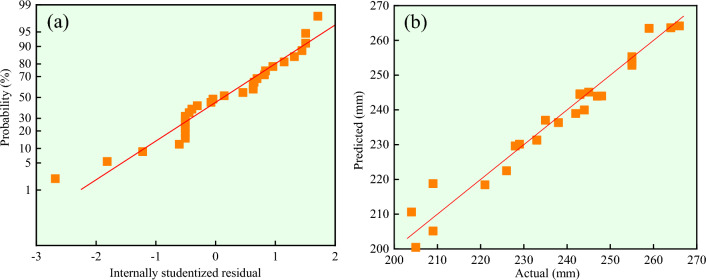
The response value *X*: (**a**) Normal plot of residuals; (**b**) Actual vs predicted.

Figure 6a,b respectively show the residual positive distribution of response value *X* and the distribution of actual and predicted values. As can be seen from the Fig. 6a, positive residual distribution, actual value and predicted value all present linear distribution. And the uniform distribution on the *y* = *x* line in Fig. 6b, which indicates that this model can be well predicted. R^2^_adj_ = 0.9375, which explains 93.75% of the variation with small errors. R^2^ = 0.9590 and C_V_ = 1.72%, indicating that the experiment is credible and accurate. Therefore, model (4) can be used to analyze and predict the response value *X*.

#### Model variance analysis of the response value *Y*

Regression analysis is performed on the response values *Y* from Table 5, and the model ANOVA results for the response values *Y* are obtained as shown in Table 7.

**Table 7:** Variance analysis results of the response value *Y.*

Source	*S*_S_	*D*_F_	*M*_S_	*F*	*P*_r_ > *F*	Note
Model	5.25	14	0.37	26.99	< 0.0001	**
*A*_1_	0.98	1	0.98	70.61	< 0.0001	**
*A*_2_	0.59	1	0.59	42.21	< 0.0001	**
*A*_3_	0.01	1	0.01	0.41	0.5312	
*A*_4_	0.05	1	0.05	3.57	0.0785	
*A*_1_*A*_2_	0.01	1	0.01	0.68	0.4209	
*A*_1_*A*_3_	0.29	1	0.29	20.81	0.0004	**
*A*_1_*A*_4_	0.04	1	0.04	3.10	0.0986	
*A*_2_*A*_3_	0.50	1	0.50	36.06	< 0.0001	**
*A*_2_*A*_4_	0.95	1	0.95	68.14	< 0.0001	**
*A*_3_*A*_4_	0.07	1	0.07	4.78	0.0451	*
*A*2 1	0.64	1	0.64	46.27	< 0.0001	**
*A*2 2	0.63	1	0.63	45.52	< 0.0001	**
*A*2 3	0.17	1	0.17	12.03	0.0034	**
*A*2 4	0.97	1	0.97	69.86	< 0.0001	**
Residual	0.21	15	0.01			
Lack of fit	0.18	10	0.02	2.95	0.1223	
Pure error	0.03	5	0.01			
Total	5.45	29				

As can be seen from Table 7, P_r_ > F < 0.000 1 is very significant, indicating that the model can be well optimized for the mix proportion. The lack of fit (P_r_ > F = 0.1223 > 0.05) is not significant, indicating that the model is significantly reliable. The F-test shows that the magnitude of the influence factor on the response value *Y* is *A*_2_ > *A*_1_ > *A*_4_ > *A*_3_*. A*_1_*A*_3_, *A*_2_*A*_3_, *A*_2_*A*_4_, *A*2 1, *A*2 2, *A*2 3 and *A*2 4 (P_r_ > F < 0.01) had a highly significant effect, *A*_3_*A*_4_ (P_r_ > F < 0.05) had a significant effect, while *A*_1_*A*_2_ and *A*_1_*A*_4_ (P_r_ > F > 0.05) had a non-significant effect.

**Figure 7:**
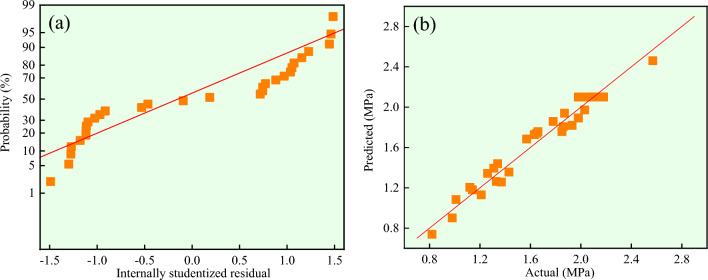
The response value *Y*: (**a**) Normal plot of residuals; (**b**) Actual vs predicted.

Figure 7a,b respectively show the positive residual distribution of response value *Y* and the distribution of actual and predicted values. As the same as the response value *X*, the positive residual distribution, actual value and predicted value of response value *Y* all present linear distribution, and also the actual and predicted values are evenly distributed along the *y* = *x* line. The correlation coefficient *R*^2^_*adj*_ is 0.9262, which explains 92.62% of the variation with small errors. R^2^ = 0.9618 and C_V_ = 7.17%, indicating that the experiment is credible and accurate. Therefore, model (5) can be used to analyze and predict the response value *Y*.

### Contour and response surface analysis

#### Contour and response surface analysis of the response value *X*

**Figure 8:**
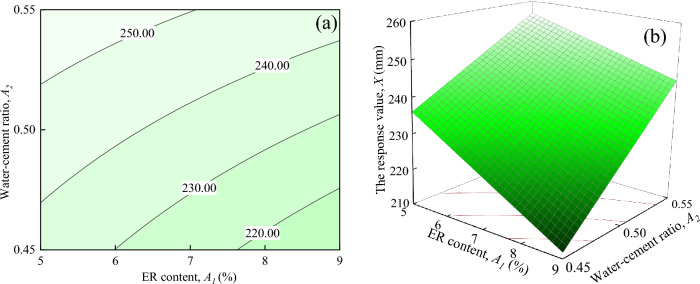
The response value *X* of the interaction between *A*_1_ and *A*_2_: (**a**) contour plot; (**b**) response surface.

**Figure 9:**
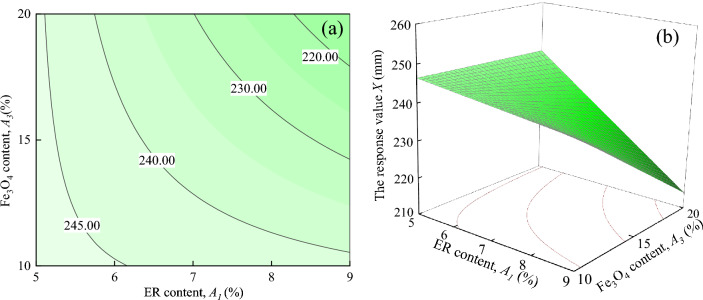
The response value *X* of the interaction between *A*_1_ and *A*_3_: (**a**) contour plot; (**b**) response surface.

**Figure 10:**
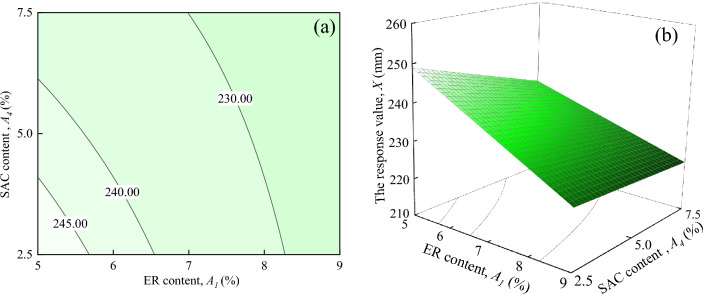
The response value *X* of the interaction between *A*_1_ and *A*_4_: (**a**) contour plot; (**b**) response surface.

**Figure 11:**
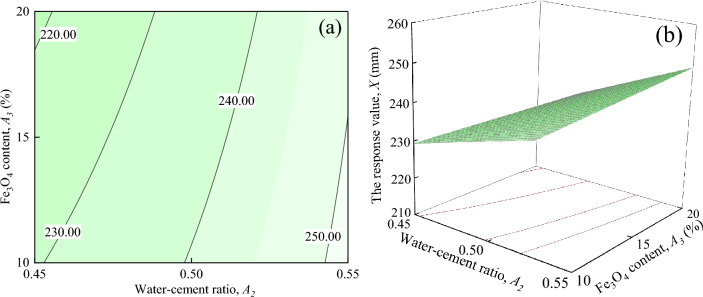
The response value *X* of the interaction between *A*_2_ and *A*_3_: (**a**) contour plot; (**b**) response surface.

**Figure 12:**
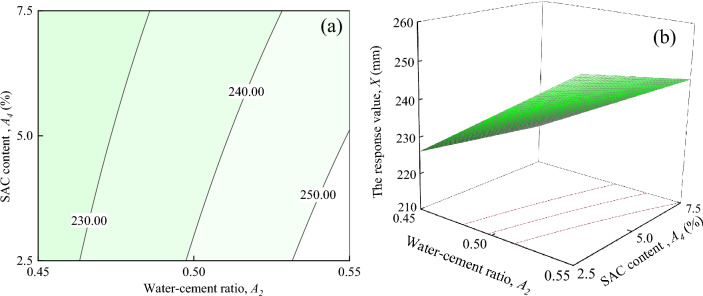
The response value *X* of the interaction between *A*_2_ and *A*_4_: (**a**) contour plot; (**b**) response surface.

**Figure 13:**
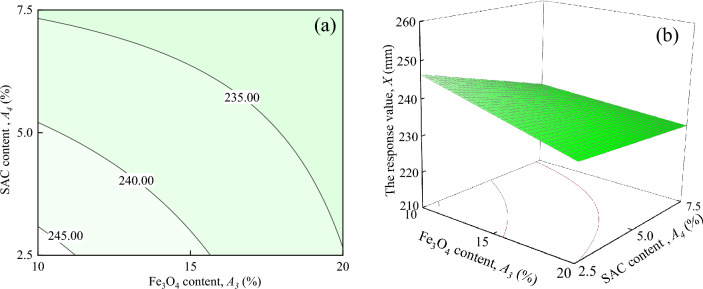
The response value *X* of the interaction between *A*_3_ and *A*_4_: (**a**) contour plot; (**b**) response surface.

The contours and response surfaces between ER content (*A*_1_), water-cement ratio (*A*_2_), Fe_3_O_4_ content (*A*_3_), SAC content (*A*_4_) and the response value *X* are shown in Figs. 8, 9, 10, 11, 12 and 13 The figure shows the influence of the interaction of the other two factors on the response value *X* when two of the four factors take a certain level.

Figure 8 illustrates the interaction effects of *ER* content, and water-cement ratio on the response value *X*, when Fe_3_O_4_ content and SAC content are located at the central level (*A*_3_ = 15%, *A*_4_ = 5%)). Figure 8a shows that the response value *X* increases as *A*_2_ increases when *A*_1_ is certain. When *A*_1_ = 7%, the response value *X* tends to change most significantly, increasing from 225.00 mm to about 250.00 mm. It can be seen from Fig. 8b that the response surface shows an overall upward trend, as *A*_1_ decreases and *A*_2_ trends up.

Figure 9 shows the interaction effects of ER content, and Fe_3_O_4_ content on the response value *X*, when the water-cement ratio and SAC content are located at the central level (*A*_2_ = 0.5, *A*_4_ = 5%). It can be seen in Fig. 9a shows that the response value *X* decreases as *A*_3_ increases when *A*_1_ is certain. When *A*_1_ = 5%, the trend of the response value *X* decreasing with *A*_*3*_ is not significant. In contrast, the trend of the response value *X* changing from *A*_1_ = 6–9% is significant. When *A*_1_ = 9%, the response value *X* drops from about 240.00 mm to 220.00 mm. It can be seen from Fig. 9b that the response surface shows a downward trend with the increase of *A*_1_ and *A*_3_.

Figure 10 displays the interaction effects of ER content, and SAC content on the response value *X*, when the water-cement ratio and Fe_3_O_4_ content are located at the central level (*A*_2_ = 0.5, *A*_3_ = 15%). As shown in Fig. 10a shows that the response value *X* decreases as *A*_4_ increases when *A*_1_ is certain. When *A*_1_ = 9%, the trend of the response value *X* decreasing with *A*_4_ is not significant. In contrast, the trend of the response value *X* changes more significantly when *A*_1_ = 5–8%. It can be seen in Fig. 10b that the response surface shows a downward trend with the increase of *A*_1_ and *A*_4_, but the trend is weaker than the interaction effect between *A*_1_ and *A*_3_.

The interaction effects of water-cement ratio and Fe_3_O_4_ content on the response value *X*, when *ER* content, and *SAC* content are located at the central level (*A*_1_ = 7%, *A*_4_ = 5%) is shown in Fig. 11. It can be seen in Fig. 11a shows that the response value *X* decreases as *A*_3_ increases when *A*_2_ is certain, but not by much. As seen in Fig. 11b that the response surface shows a slow upward trend with the increase of *A*_2_ and *A*_3_.

The Fig. 12 demonstrates the interaction effects of water-cement ratio and SAC content on the response value *X*, when ER content, and Fe_3_O_4_ content are located at the central level (*A*_1_ = 7%, *A*_3_ = 15%). The Fig. 12a shows that the response value *X* decreases as *A*_4_ increases when *A*_2_ is certain, but not by much. It can be seen in Fig. 12b that the response surface shows a slow upward trend with the increase of *A*_2_ and *A*_4_. This change trend is similar to the interaction effect between *A*_2_ and *A*_3_.

Figure 13 shows the interaction effects of SAC content and Fe_3_O_4_ content on the response value *X*, when ER content, and water-cement ratio are located at the central level (*A*_1_ = 7%, *A*_2_ = 0.5). It can be seen from Fig. 13a shows that the response value *X* decreases as *A*_4_ increases when *A*_3_ is certain. When *A*_3_ = 10%, the response value *X* tends to change more significantly, decreasing from about 245.00 mm to 230.00 mm. While *A*_3_ = 20%, the response value *X* tends to change insignificantly with *A*_4_. As shown in Fig. 13b shows that the response surface shows an overall increasing trend with the increase of *A*_3_ and *A*_4_.

As shown in Figs. 8, 9, 10, 11, 12 and 13, a comprehensive comparison of the factors' interactions on the response value X reveals no significant interaction between *A*_*2*_ and *A*_*3*_, or *A*_*2*_ and *A*_*4*_. However, the interaction between *A*_*1*_ and *A*_*3*_, and *A*_*1*_ and *A*_*2*_ are more significant than the interaction between *A*_*1*_ and *A*_*4*_, and *A*_*3*_ and *A*_*4*_. Combined with Table 6, it can be seen that the degree of interaction between the factors on the response value *X* in the ascending order of *A*_*1*_*A*_*3*_ > *A*_*1*_*A*_*2*_ > *A*_*1*_*A*_*4*_ > *A*_*3*_*A*_*4*_ > *A*_*2*_*A*_*3*_ > *A*_*2*_*A*_*4*_.

#### Contour and response surface analysis of the response value *Y*

**Figure 14:**
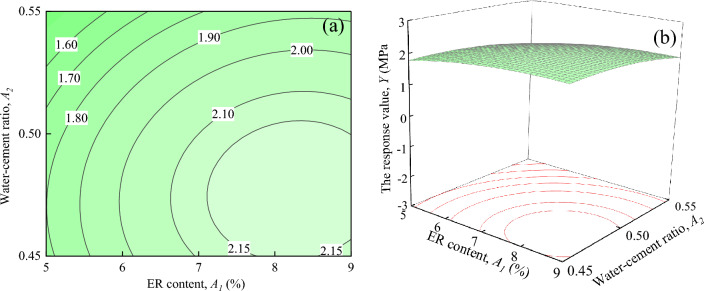
The response value *Y* of the interaction between *A*_*1*_ and *A*_*2*_: (**a**) contour plot; (**b**) response surface.

**Figure 15:**
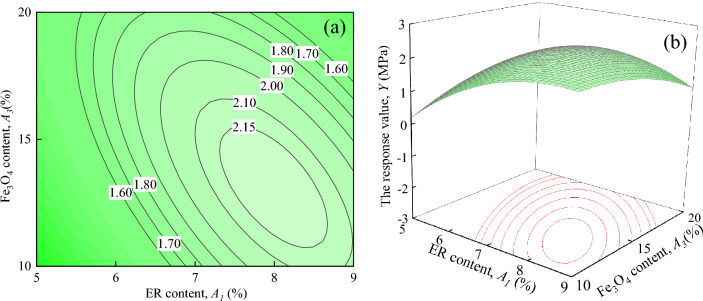
The response value *Y* of the interaction between *A*_*1*_ and *A*_*3*_: (**a**) contour plot; (**b**) response surface.

**Figure 16:**
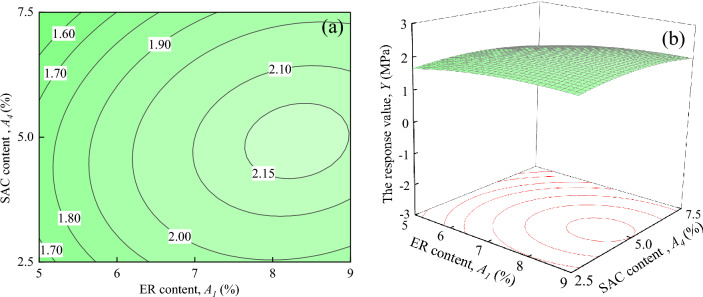
The response value *Y* of the interaction between *A*_*1*_ and *A*_*4*_: (**a**) contour plot; (**b**) response surface.

**Figure 17:**
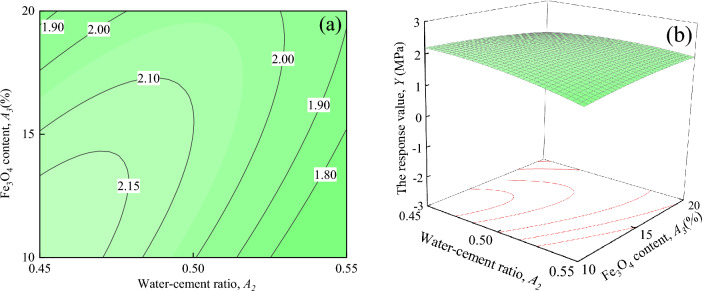
The response value *Y* of the interaction between *A*_*2*_ and *A*_*3*_: (**a**) contour plot; (**b**) response surface.

**Figure 18:**
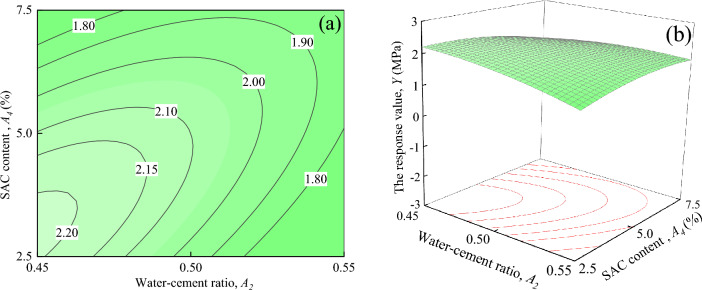
The response value *Y* of the interaction between *A*_*2*_ and *A*_*4*_: (**a**) contour plot; (**b**) response surface.

**Figure 19:**
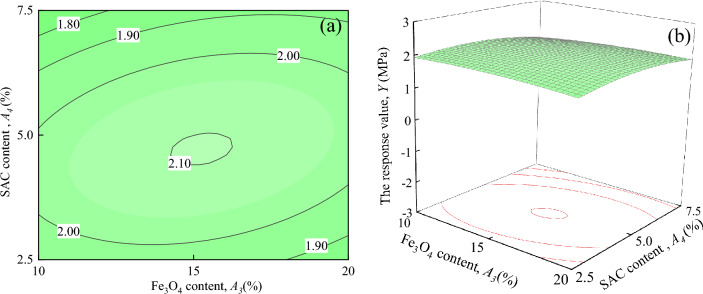
The response value *Y* of the interaction between *A*_*3*_ and *A*_*4*_: (**a**) contour plot; (**b**) response surface.

The contours and response surfaces between ER content (*A*_*1*_), water-cement ratio (*A*_*2*_), Fe_3_O_4_ content (*A*_*3*_), SAC content (*A*_*4*_) and the response value *Y* are shown in Fig. 14, 15, 16, 17, 18 and 19.

Figure 14 shows the interaction effects of ER content, and water-cement ratio on the response value *Y*, when Fe_3_O_4_ content and SAC content are located at the central level (*A*_*3*_ = 15%, *A*_*4*_ = 5%). It can be seen from Fig. 14a that the shape of the contours is circular^29^. This indicates that the interaction between *A*_*1*_ and *A*_*2*_ is not significant, which is consistent with the results in Table 7. It can be seen in Fig. 14b shows that the slope of the response surface is very gentle in the direction of *A*_*1*_ and *A*_*2*_ changes. Basically no surface changes can be found, indicating that *A*_*1*_ and *A*_*2*_ have limited influence on the response value *Y*.

Figure 15 illustrates the interaction effects of ER content, and Fe_3_O_4_ content on the response value *Y*, when the water-cement ratio and SAC content are located at the central level (*A*_*2*_ = 0.5, *A*_*4*_ = 5%). As seen in Fig. 15a, the shape of the contours is elliptical^29^, indicating that the interaction between *A*_*1*_ and *A*_*3*_ is significant, which is consistent with the results in Table 7. It can be seen in Fig. 15b that with an increase in *A*_*1*_ and *A*_*3*_, the response surface shows a tendency to rise and then fall, exhibiting an upward convex spherical surface.

Figure 16 demonstrates the interaction effects of ER content, and SAC content on the response value *Y*, when the water-cement ratio and Fe_3_O_4_ content are located at the central level (*A*_*2*_ = 0.5, *A*_*3*_ = 15%). As seen in Fig. 16a that the contours exhibit elliptical characteristics, indicating a significant interaction between *A*_*1*_ and *A*_*4*_. It can be seen in Fig. 16b shows that the slope of the response surface is gentler in the direction of change of *A*_*1*_ and *A*_*4*_ This indicates that *A*_*1*_ and *A*_*4*_ have some influence on the response value *Y*. However, this influence is not very significant, which is consistent with the results in Table 7.

Figure 17 displays the interaction effects of water-cement ratio and Fe_3_O_4_ content on the response value *Y*, when ER content, and SAC content are located at the central level (*A*_*1*_ = 7%, *A*_*4*_ = 5%). As seen in Fig. 17a that the response value *Y* decreases as *A*_*3*_ increases when *A*_*2*_ is certain. When *A*_*2*_ = 0.55, the trend of the response value *Y* does not change significantly. However, the trend of the response value *Y* changes significantly when *A*_*2*_ = 0.45, decreasing from about 2.15 to 1.90 MPa. It can be seen from Fig. 17b that the response surface shows a downward trend, as *A*_*2*_ falls and *A*_*3*_ increases.

Figure 18 shows the interaction effects of water-cement ratio and SAC content on the response value *Y*, when ER content, and Fe_3_O_4_ content are located at the central level (*A*_*1*_ = 7%, *A*_*3*_ = 15%). It can be seen in Fig. 18a that when *A*_*2*_ is certain, the response value *Y* decreases with the increase of *A*_*4*_. When *A*_*2*_ = 0.55, the trend of change in the response value *Y* is not significant. However, the trend of the response value *Y* changes significantly when *A*_*2*_ = 0.45 ~ 0.50, and decreases from 2.20 MPa to about 1.80 MPa when *A*_*2*_ = 0.45. It can be seen from Fig. 18b that the response surface shows a downward trend with the increases of *A*_*2*_ and *A*_*4*_.

Figure 19 shows the interaction effects of SAC content and Fe_3_O_4_ content on the response value *Y*, when ER content, and water-cement ratio are located at the central level (*A*_*1*_ = 7%, *A*_*2*_ = 0.5). As seen in Fig. 19a, the shape of the contours is elliptical, indicating that the interaction between *A*_*3*_ and *A*_*4*_ is significant, which is consistent with the results in Table 7. It can be seen in Fig. 19b that with an increase in *A*_*3*_ and *A*_*4*_, the response surface shows a tendency to rise and then fall, exhibiting an upward convex spherical surface.

As shown in Figs. 14, 15, 16, 17, 18 and 19, a comprehensive comparison of the factors' interactions on the response value *Y* reveals no significant interaction between *A*_*1*_ and *A*_*2*_, or *A*_*1*_ and *A*_*4*_. However, the interaction between *A*_*2*_ and *A*_*3*_, and *A*_*2*_ and *A*_*4*_ are more significant than the interaction between*A*_*1*_ and *A*_*3*_*,* and *A*_*3*_ and *A*_*4*_. Combined with Table 7, it can be seen that the degree of interaction between the factors on the response value *Y* in the ascending order of *A*_*2*_*A*_*3*_ > *A*_*2*_*A*_*4*_ > *A*_*1*_*A*_*3*_ > *A*_*3*_*A*_*4*_ > *A*_*1*_*A*_*4*_ > *A*_*1*_*A*_*2*_.

### Optimal mix proportion and model verification

The optimal combination of factors obtained by Design Expert optimization analysis is: 8.78% ER content, 0.45water-cement ratio, 10% Fe_3_O_4_ content, 2.96% SAC content. The predicted values of the *X* and *Y* are 223.31 mm and 2.47 MPa, respectively, and the corresponding evaluation index *F* is 551.58. In order to facilitate the experimental operation, the optimal mix proportions were adjusted as follows: 8.8% ER content, 0.45 water-cement ratio, 10% Fe_3_O_4_ content, 3.0% SAC content, and other conditions are the same as the basic mix proportions.

To verify the reliability of the model, the above optimal fit magnetically driven epoxy resin cement slurry was formulated and the results are shown in Table 8. It can be seen from Table 8 that the correlation between the predicted value and the actual value is high. The *X*, *Y* and *F* are 222.50 mm, 2.43 MPa and 540.68 respectively. The relative error is only 0.36%, 1.65% and 2.02%.

**Table 8:** Comparison of predictors and real values in an optimization model.

Factor				Predicted value			Actual value			Relative error (%)		
*A*_*1*_ (%)	*A*_*2*_	*A*_*3*_ (%)	*A*_*4*_ (%)	*X* (mm)	*Y* (MPa)	*F*	*X* (mm)	*Y* (MPa)	*F*	*X*	*Y*	*F*
8.8	0.45	10	3	223.31	2.47	551.58	222.50	2.43	540.68	0.36	1.65	2.02

## Microscopic characterization of optimal mix proportion

### Phase composition

**Figure 20:**
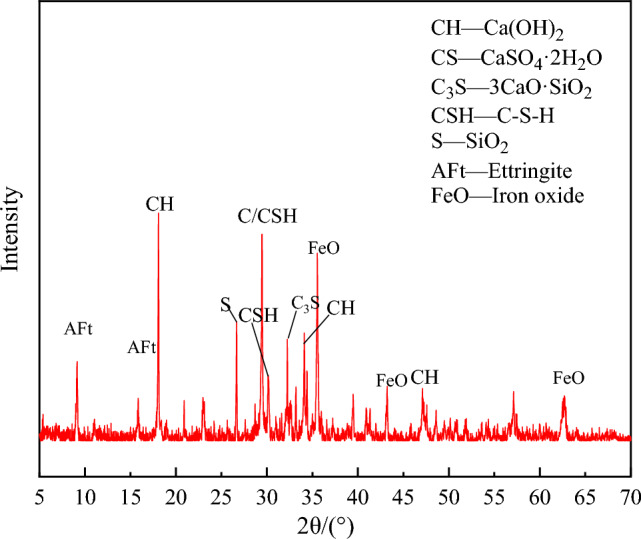
The phase composition of solidification at 28 days.

Figure 20 displays the XRD test results of solidification. As can be seen in Fig. 20, the strongest diffraction peak of ettringite is 2θ = 9.1°, the strongest diffraction peak of iron oxide is 2θ = 35.5°, the strongest diffraction peak of hydrated calcium silicate is 2θ = 29.5°, the strongest diffraction peak of tricalcium silicate is 2θ = 32.2°, the strongest diffraction peak of Ca(OH)_2_ is 2θ = 18.0°, and the strongest diffraction peak of SiO_2_ is 2θ = 26.6°. The cement in solidification is fully hydrated, and a large amount of C-S–H gel and ettringite are formed.

### Phase characteristics

**Figure 21:**
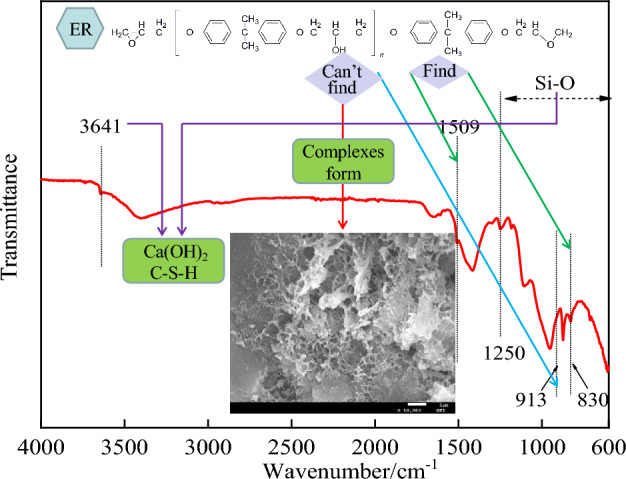
The phase characteristics of solidification at 28 days.

In Fig. 21, the physical phase characteristics of solidification can be seen. As seen in Fig. 21, the solidification has the flat and compact morphology of a typical complex. The epoxy resin cured material interacts ionically with Ca^2+^ in AFt and Ca(OH)_2_ to form complexes, and a large amount of crystalline material is generated^13^. The characteristic peak of epoxy resin is present at 830 cm^−1^^30^. 1509 cm^−1^ is the peak of C=C bond stretching vibration in the benzene ring^31^, which is the characteristic peak of ER. There is no epoxy group vibration peak at 913 cm^−1^, indicating that ER can open the ring smoothly in the cement base and complete the curing process^30,31^. The area range 1250–600 cm^−1^ is the region of Si–O characteristic peaks^32^, which are the relevant characteristic peaks of hydrated calcium silicate (C–S–H) products. 3641 cm^−1^ is the Ca-OH vibrational absorption peak, which proves the presence of Ca(OH)_2_ in the hydration product and is consistent with the XRD test results.

## Conclusion

1. This paper proposes to evaluate the index *F*, which can effectively evaluate the interaction between the fluidity and tensile strength of the slurry.
2. The 2FI regression model and the quadratic regression model are developed with fluidity and tensile strength as response values. In addition, ER content, water-cement ratio, Fe_3_O_4_ content and SAC content are considered influencing factors. ANOVA and model fit tests validated the models, and the 2 regression models have reasonable fit and reliability.
3. According to the ANOVA, the relationship between the degree of influence of the influencing factors on response value fluidity (*X*) and tensile strength (*Y*) in ascending order is: ER content > water-cement ratio > SAC content > Fe_3_O_4_ content.
4. When the 8.8% ER content, 0.45 water-cement ratio, 10% Fe_3_O_4_ content, and 3.0% SAC content, the response value *X* is 223.31 mm, the response value *Y* is 2.47 MPa, and the corresponding evaluation index *F* is 551.58, with relative errors of only 0.36%, 1.65%, and 2.02% respectively, indicating that the regression model fits well and the parameters are reliable.
5. The XRD, SEM and FTIR analyses show that the magnetically driven epoxy resin cement slurry is well hydrated, generating a large number of C–S–H gels and calcium alumina generation, and ER can be cured smoothly with the flat and compact morphology of a typical complex.

## Data Availability

The authors confirm that the data supporting the findings of this study are available within the article.

## References

[1] Tang SW, Yao Y, Andrade C, Li ZJ (2015). Recent durability studies on concrete structure. Cement Concr. Res..

[2] Wei Y, Guo W, Wu Z, Gao X (2020). Computed permeability for cement paste subject to freeze-thaw cycles at early ages. Constr. Build. Mater..

[3] Chen Z, Wu L, Bindiganavile V, Yi C (2020). Coupled models to describe the combined diffusion-reaction behaviour of chloride and sulphate ions in cement-based systems. Constr. Build. Mater..

[4] Chen Y, Gao J, Tang L, Li X (2016). Resistance of concrete against combined attack of chloride and sulfate under drying-wetting cycles. Constr. Build. Mater..

[5] Ren J, Guo SY, Su J, Zhao TJ, Chen JZ, Zhang SL (2019). A novel TiO_2_/Epoxy resin composited geopolymer with great durability in wetting-drying and phosphoric acid solution. J. Cleaner Prod..

[6] Maes M, De Belie N (2014). Resistance of concrete and mortar against combined attack of chloride and sodium sulphate. Cem. Concr. Compos..

[7] Cheng S, Shui Z, Gao X, Yu R, Sun T, Guo C, Huang Y (2020). Degradation mechanisms of Portland cement mortar under seawater attack and drying–wetting cycles. Constr. Build. Mater..

[8] Cho CG, Kim YY, Feo L, Hui D (2012). Cyclic responses of reinforced concrete composite columns strengthened in the plastic hinge region by HPFRC mortar. Compos. Struct..

[9] Do J, Soh Y (2003). Performance of polymer-modified self-leveling mortars with high polymer-cement ratio for floor finishing. Cem. Concr. Res..

[10] Aggarwal LK, Thapliyal PC, Karade SR (2007). Properties of polymer-modified mortars using epoxy and acrylic emulsions. Constr. Build. Mater..

[11] Smoak, W. G. *Guide to Concrete Repair, US Department of the Interior, Bureau of Reclamation* (Technical Service Center, 1997).

[12] Feng, Z. L., Li, Y. Nano Fe_3_O_4_ modified epoxy resin and its application in underwater in-situ repair of organic coating defects. In *11th National Conference on Corrosion and Protection, Shenyang, Liaoning, China* (2021).

[13] Jithendra Reddy C (2017). Geopolymer concrete with self compacting: A review. Int. J. Civ Eng. Technol..

[14] EFNARC. Specification and Guidelines for Self-Compacting Concrete, Rep. from EFNARC **44**, 32 (2002).

[15] Nagaraj VK, Babu DLV (2018). Assessing the performance of molarity and alkaline activator ratio on engineering properties of self-compacting alkaline activated concrete at ambient temperature. J. Build. Eng..

[16] Liu J, Li Z, Li Z, Sun T, Chen QF, Qin SF (2023). Hardening mechanism and pore size analysis of new magnetic epoxy cement grout. Acta Mater. Compositae Sin..

[17] Zhang C, Yang JS, Xie YP, Gong FH, Liang X, Lei JS, Su BZ (2018). Experiment and application for grouting materials for karst under conditions of underground water flow before shield tunneling. Chin. J. Rock Mech. Eng..

[18] Tayeh BA, Bakar BHA, Johari MAM, Voo YL (2013). Evaluation of bond strength between normal concrete substrate and ultra high performance fiber concrete as a repair material. Proc. Eng..

[19] Guo SY, Zhang X, Ren J, Chen JZ, Zhao TJ, Li TW, Zhang LH (2021). Preparation of TiO_2_/epoxy resin composite and its effect on mechanical and bonding properties of OPC mortars. Constr. Build. Mater..

[20] Myers RH, Montgomery DC (2002). Response Surface Methodology: Process and Product Optimization Using Designed Experiments.

[21] Mohammed BS, Khed VC, Nuruddin MF (2018). Rubbercrete mixture optimization using response surface methodology. J. Clean. Prod..

[22] Danmalik IGI, Saleh TA, Shamsuddeen AA (2017). Response surface methodology optimization of adsorptive desulfurization on nickel/activated carbon. Chem. Eng. J..

[23] Momayez A, Ehsani MR, Ramezanianpoura AA, Rajaiea H (2005). Comparison of methods for evaluating bond strength between concrete substrate and repair materials. Cem. Concr. Res..

[24] Ren J, Guon SY, Zhao TJ, Chen JZ, Nicolas RS, Zhang LH (2020). Constructing a novel nano-TiO_2_/Epoxy resin composite and its application in alkali-activated slag/fly ash pastes. Constr. Build. Mater..

[25] Ghannam S, Najm H, Vasconez R (2016). Experimental study of concrete made with granite and iron powders as partial replacement of sand. Sustain. Mater. Technol..

[26] Wang X, Bai XM, Liu C, Jiang LZ, Xiao ZM (2004). On influence of particle shape of cement on its property. J. Chin. Ceram. Soc..

[27] Xu, X. W. *The Experimental Study on Properties of Portland Cement-Sulphoaluminate Cement Composite System.* (Shenyang Jianzhu University, 2018).

[28] Li R, Jiang YH, Zhang YL, Chen KQ, Huang BF, Li L (2022). Optimisation of ammonium sulphate crystallization based on response surface methodology. J. Chin. Ceram. Soc..

[29] Liu YH, Chen TH, Wang C, Liu HB, Chen D, Zou XH, Xie JJ (2020). 4A zeolite derived from weathered washing sand tail mud as a high-efficiency NH_4_^+^-N adsorbent. J. Chin. Ceram. Soc..

[30] Li SY, Wang YL, Liu ZY, Hou LJ (2019). Effects of curing condition and polymer-cement ratio on properties of toughened waterborne epoxy cement-based coatings. J. Chin. Ceram. Soc..

[31] Zhou HR, Mao SS (2018). Preparation and properties of nano SiO_2_-carboxyl carboxyl nitrile rubber modified water-based epoxy resin composite. Acta. Mate. Compositae. Sin..

[32] Zhu XH, Li Q, Kang XJ, Deng JX, Yang K (2021). Nano-structural change of C(N)-A-S-H gel in alkali-activated slag pastes subjected to wetting-drying cyclic sulphate attack. J. Chin. Ceram. Soc..

